# Metabolomic and lipidomic signatures associated with clinical improvement after balloon pulmonary angioplasty in chronic thromboembolic pulmonary hypertension

**DOI:** 10.1016/j.pccm.2026.02.007

**Published:** 2026-03-10

**Authors:** Jinglin Tian, Sugang Gong, Ping Yuan, Jingru Huang, Xincheng Li, Jun Wang, Tao Wang, Zhenchi Li, Lan Wang, Deming Gou

**Affiliations:** a Vascular Disease Research Center, College of Life Sciences and Oceanography, Shenzhen University, Shenzhen, Guangdong 518055, China; b Department of Pulmonary Circulation, Shanghai Pulmonary Hospital, School of Medicine, Tongji University, Shanghai 200433, China; c Department of Rehabilitation Medicine, Shanghai Pulmonary Hospital, School of Medicine, Tongji University, Shanghai 200433, China; d School of Pharmacy, Shenzhen University Medical School, Shenzhen University, Shenzhen, Guangdong 518055, China

Dear Editor,

Chronic thromboembolic pulmonary hypertension (CTEPH) is a unique and potentially reversible subtype of pulmonary hypertension characterized by persistent thromboembolic obstruction of the pulmonary vasculature, progressive pulmonary vascular remodeling, and right ventricular dysfunction. Pulmonary endarterectomy remains the treatment of choice for operable disease; however, a substantial proportion of patients are deemed inoperable or develop residual pulmonary hypertension following surgery. For these patients, balloon pulmonary angioplasty (BPA) has emerged as a guideline-recommended first-line therapeutic strategy, with growing evidence supporting its efficacy in improving pulmonary hemodynamics, exercise capacity, and clinical outcomes.^1^^,^^2^

While the hemodynamic and functional benefits of BPA are well established, its systemic biological consequences remain incompletely understood. How mechanical relief of pulmonary vascular obstruction translates into coordinated metabolic recovery at the systemic level has not been fully elucidated. Circulating metabolites integrate signals from pulmonary vascular remodeling, right ventricular load, hypoxia, inflammation, and mitochondrial function, and thus provide a promising, non-invasive window into disease reversibility and treatment response.^3^ However, comprehensive metabolomic and lipidomic characterization of BPA-associated changes in CTEPH has been limited.

To address this gap, we performed a prospective, paired plasma metabolomic and lipidomic analysis in patients with CTEPH undergoing BPA, with independent validation using targeted metabolomics (Fig. 1A). Detailed methods for sample collection and metabolomics analysis are provided in the Supplementary Methods. The discovery cohort comprised 65 consecutive patients treated between January 2020 and May 2023, in whom plasma samples were collected before BPA and after completion of angiographically confirmed revascularization of all targeted pulmonary artery segments. An independent external cohort of 17 patients treated between May 2023 and October 2024 was included for targeted validation. All patients fulfilled contemporary guideline-based diagnostic criteria and underwent standardized clinical assessment including right heart catheterization, echocardiography, and 6-minute walk distance (6MWD) testing. This study was approved by the Ethics Committee of Shanghai Pulmonary Hospital (No. K23-022Z) and followed the *Declaration of Helsinki*. All participants provided written informed consent.

**Fig. 1:**
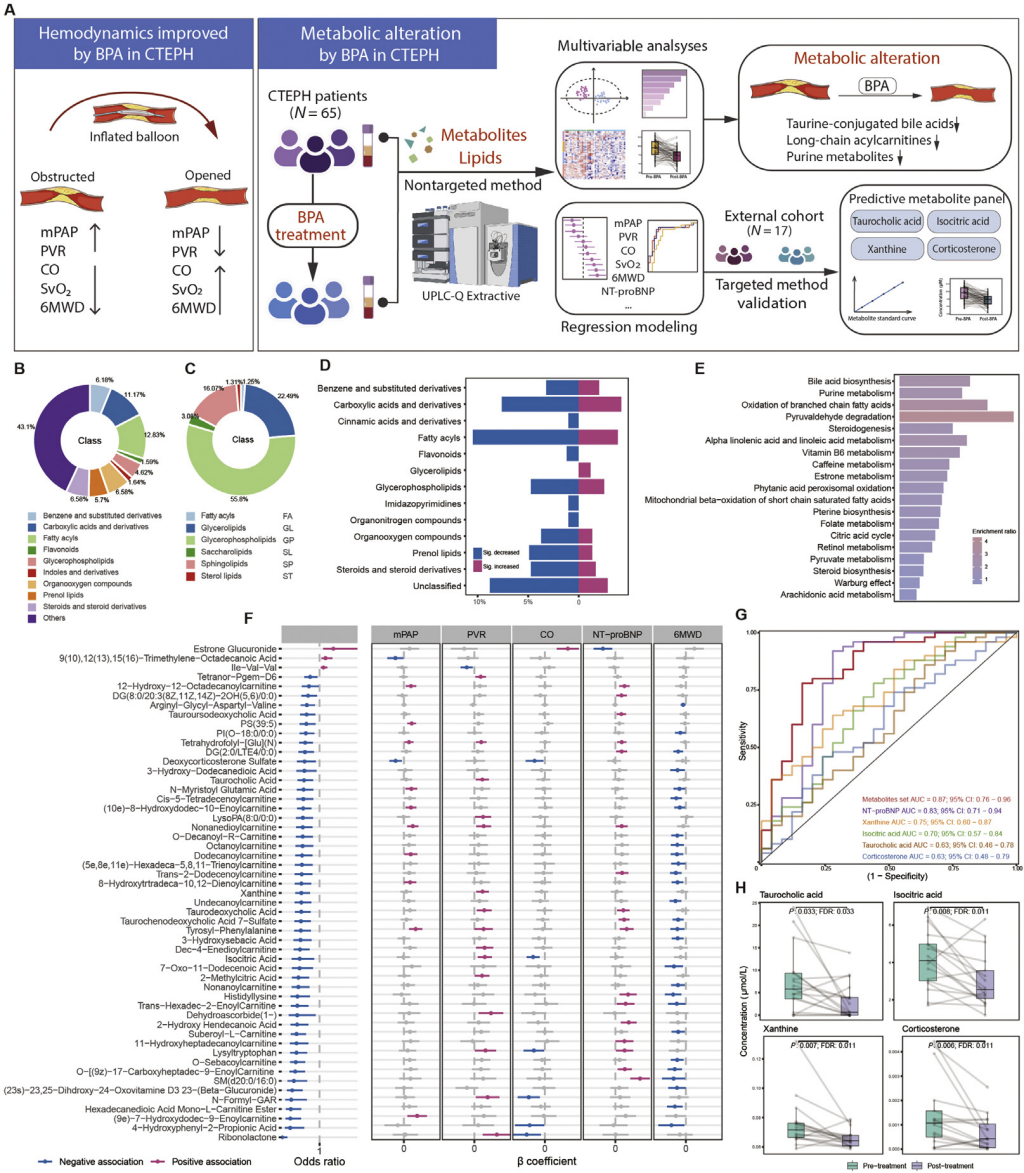
Hemodynamic improvements and metabolic alterations induced by balloon pulmonary angioplasty (BPA) in patients with chronic thromboembolic pulmonary hypertension (CTEPH). (A) Schematic overview of the study design and key findings. (B–C) Pie charts illustrating the relative distribution of metabolite/lipid classes identified in the dataset. (D) Bar plot depicting the direction and magnitude of changes in major metabolite classes following BPA treatment. Blue bars indicate significantly decreased metabolites, and purple bars indicate significantly increased metabolites. (E) Pathway enrichment analysis of differentially altered metabolites post-BPA. (F) Associations between selected metabolites and clinical/hemodynamic parameters. Left: forest plot showing odds ratios for binary outcomes or associations. Right: β coefficients for continuous variables. Blue symbols denote negative associations; purple symbols denote positive associations. (G) Receiver operating characteristic curves evaluating the predictive performance of metabolite panels for hemodynamic or clinical improvement. (H) Box plots illustrating pre- and post-BPA concentrations of key validated metabolites in the validation cohort. Statistical significance is indicated, with lines connecting paired samples where applicable. AUC, Area under the curve; 6MWD, 6-Minute walk distance; CO, Cardiac output; FDR, False discovery rate; mPAP, Mean pulmonary artery pressure; NT-proBNP, N-terminal pro-B-type natriuretic peptide; PVR, Pulmonary vascular resistance; SvO2, Mixed venous oxygen saturation; UPLC, Ultra-high pressure liquid chromatography.

Detailed information regarding data completeness for clinical and metabolomic variables is provided in Supplementary Table 1. The demographics and clinical characteristics of all CTEPH participants undergoing BPA are summarized in Supplementary Tables 2 and 3. Consistent findings were observed in paired clinical comparisons, showing significant hemodynamic and functional improvement in both the discovery cohort and the validation cohort (Supplementary Figs. 1 and 2). Mean pulmonary artery pressure and pulmonary vascular resistance decreased, while cardiac output (CO), mixed venous oxygen saturation, and 6MWD increased, accompanied by a reduction in N-terminal pro–B-type natriuretic peptide (NT-proBNP). These findings are consistent with prior randomized and real-world studies demonstrating the clinical efficacy of BPA in inoperable CTEPH.^2^

Beyond hemodynamic improvement, integrated untargeted metabolomic and lipidomic profiling revealed marked systemic metabolic remodeling following BPA (Fig. 1A, middle and right panels). Multivariate analyses demonstrated clear separation between pre- and post-treatment samples, indicating that BPA induces a coordinated shift in the circulating metabolic landscape rather than isolated changes in individual metabolites. The robustness of pre- and post-treatment discrimination by partial least squares discriminant analysis (PLS-DA) and orthogonal partial least squares discriminant analysis (OPLS-DA) was supported with 200-permutation testing (Supplementary Fig. 3).

Global classification of detected metabolites and lipids revealed distinct compositional changes after BPA. At the class level, fatty acyls, glycerophospholipids, and sphingolipids accounted for a substantial proportion of metabolites (Fig. 1B and C). Comparative analysis of metabolite subclasses demonstrated a preferential reduction in fatty acyls and steroid derivatives, alongside selective increases in specific glycerophospholipid species (Fig. 1D). These findings suggest that BPA induces broad reprogramming of lipid metabolism, potentially reflecting improved mitochondrial function and altered substrate utilization following right ventricular unloading.

Pathway enrichment analysis further highlighted bile acid biosynthesis, purine metabolism, and fatty acid β-oxidation as the most prominently altered metabolic pathways after BPA (Fig. 1E). Among these, bile acid metabolism emerged as a key feature of post-BPA metabolic remodeling. Taurine-conjugated bile acids, including taurocholic acid and taurodeoxycholic acid, were consistently and significantly reduced after treatment (Fig. 1F). Emerging evidence indicates that aberrant bile acid metabolism contributes to pulmonary vascular endothelial dysfunction, inflammation, and endoplasmic reticulum stress in pulmonary hypertension.^4^ The observed reduction in taurine-conjugated bile acids may therefore reflect attenuation of systemic inflammatory and cellular stress responses following restoration of pulmonary blood flow. The Kyoto Encyclopedia of Genes and Genomes (KEGG)-based enrichment analyses were also performed for metabolites and lipids, respectively (Supplementary Figs. 4 and 5).

In parallel, multiple medium- and long-chain acylcarnitines were decreased after BPA and exhibited strong associations with improvements in pulmonary hemodynamics, NT-proBNP, and 6MWD (Fig. 1F). Accumulation of acylcarnitines is a recognized marker of impaired mitochondrial fatty acid oxidation and right ventricular lipotoxicity, both of which have been implicated in maladaptive cardiac remodeling and heart failure in pulmonary hypertension.^5^ The reduction in circulating acylcarnitines following BPA suggests partial normalization of mitochondrial metabolic efficiency accompanying hemodynamic unloading.

Conversely, several phosphatidylcholine species, including omega-3 polyunsaturated fatty acid-containing phosphatidylcholines and plasmalogen phosphatidylcholines, were increased after BPA (Fig. 1D). Disrupted phospholipid metabolism has previously been linked to disease severity and adverse outcomes in both CTEPH and pulmonary arterial hypertension.^3^^,^^6^ The selective enrichment of these lipid species may reflect restoration of membrane lipid homeostasis and a shift toward a more favorable cardiometabolic profile following effective mechanical relief of pulmonary vascular obstruction.

To integrate metabolic changes with clinical outcomes, we performed combined conditional logistic regression and multivariable linear regression analyses incorporating hemodynamic and functional parameters. The comprehensive analytical framework further indicated that many of these metabolites were simultaneously associated with BPA treatment status and standardized changes in key clinical indicators (Supplementary Fig. 6). This approach identified 81 endogenous metabolites and lipids significantly associated with BPA treatment status and clinical improvement. Their levels change is further illustrated in a heatmap overview (Supplementary Fig. 7). The forest plot visualized associations between the differentially abundant metabolites and five of key clinical parameters, including cardiac output (CO), mean pulmonary artery pressure (mPAP), pulmonary vascular resistance (PVR), NT-proBNP and 6MWD, with significant associations highlighted in color (Fig. 1F). Notably, several metabolites demonstrated consistent associations across multiple clinical indices, supporting their potential role as integrative markers of disease severity and recovery.

From these analyses, a parsimonious four-metabolite panel—taurocholic acid, isocitric acid, xanthine, and corticosterone—was derived and demonstrated robust discriminatory performance for distinguishing post- from pre-BPA states (area under the receiver operating characteristic curve [AUC] = 0.87), outperforming NT-proBNP alone (Fig. 1G). Importantly, targeted metabolomic analyses in the independent validation cohort confirmed significant and directionally consistent reductions in all four metabolites after BPA (Fig. 1H), supporting the reproducibility and translational potential of this biomarker panel. Mechanistically, downregulation of purine metabolites such as xanthine is consistent with alleviation of tissue hypoxia, oxidative stress, and maladaptive metabolic demand—processes closely linked to pulmonary vascular remodeling and right ventricular dysfunction.^7^ Corticosterone reduction may further reflect attenuation of neurohormonal and stress-related activation following hemodynamic improvement. Together, these coordinated metabolic changes suggest that BPA induces not only mechanical and hemodynamic benefits but also systemic metabolic recovery.

Several limitations warrant consideration. This was a single-center study with a modest sample size, and its observational design precludes definitive causal inference. Additionally, longer-term prognostic implications of the identified metabolic signatures remain to be established. Nonetheless, the consistency between discovery and validation cohorts and the close coupling between metabolic remodeling and clinical improvement support the biological relevance of our findings.

In conclusion, our study demonstrates that BPA in CTEPH is associated with coordinated improvement in pulmonary hemodynamics, functional capacity, and systemic metabolism. Integrated metabolomic and lipidomic profiling reveals the alterations in bile acid, purine, and fatty acid metabolism, and identifies a circulating metabolite panel with potential utility for non-invasive monitoring of treatment response. These findings provide new insight into the systemic biological effects of BPA and highlight circulating metabolites as integrative markers of disease reversibility in CTEPH.

## Funding

This work was supported by grants from Non-communicable Chronic Diseases-National Science and Technology Major Project (No. 2024ZD0526700), Shenzhen Medical Research Fund (No. C2404001 and B2302015), Program of National Key Research and Development Plan (No. 2024ZD0528600), National Natural Science Foundation of China (Nos. 82400070, 82370057, 82241022, 82470056 and 82200067) and the Program of Natural Science Foundation of Shanghai (No. 22ZR1452400).

## CRediT authorship contribution statement

**Jinglin Tian:** Writing – original draft, Visualization, Formal analysis, Conceptualization. **Sugang Gong:** Methodology, Investigation, Data curation. **Ping Yuan:** Resources, Project administration. **Jingru Huang:** Visualization. **Xincheng Li:** Writing – review & editing. **Jun Wang:** Conceptualization. **Tao Wang:** Investigation. **Zhenchi Li:** Visualization, Software, Methodology, Formal analysis, Conceptualization. **Lan Wang:** Writing – review & editing, Supervision. **Deming Gou:** Supervision, Funding acquisition.

## Declaration of competing interest

The authors declare that they have no known competing financial interests or personal relationships that could have appeared to influence the work reported in this paper.

## Appendix Supplementary materials

Image, application 1
