# Supplementary material for: Metabolomic and lipidomic signatures associated with clinical improvement after balloon pulmonary angioplasty in chronic thromboembolic pulmonary hypertension
Source: Chin Med J Pulm Crit Care Med. 2026 Mar 10;4(1):92–4. doi: 10.1016/j.pccm.2026.02.007 (PMC13063257; doi:10.1016/j.pccm.2026.02.007)
Supplement: Supplementary file 1 [file mmc1.docx]

**Supplementary Methods**

**Plasma Sample Collection**

Plasma sampling was performed usually 1 to 3 days before BPA surgery in patients recruited at Shanghai Pulmonary Hospital. To prevent activation of the coagulation system, all samples were collected in vacutainer tubes containing ethylene diamine tetraacetic acid (EDTA) as an anticoagulant. The blood samples were then centrifuged (1300 g, 15 min) to separate plasma from the cellular components. The plasma was carefully transferred to cryovials, avoiding any contact with the cell pellet, and stored at -80°C until further analysis. Plasma samples were thawed only once immediately before the assays were performed to preserve sample integrity.

**Non-targeted Metabolomics and Lipidomics Analysis**

***Sample preparation for LC-MS/MS detection***

The plasma samples were thawed in an ice-water mixture. Subsequently, samples prepared for metabolomics and lipidomics were prepared respectively. All reagents were pre-cooled before use. The 100 μL of plasma was mixed with 400 μL methanol, acetonitrile, and ultra-pure water (in a 6:3:1 ratio), containing a mixed internal standard (L-2-chlorophenylalanine, succinic acid-D4, L-valine-D8), was then added to the plasma to precipitate the proteins. After vortexing for 1 minute, ultrasonic extraction was conducted in an ice water bath for 10 minutes, followed by incubation at -40℃ for 30 minutes. The samples were then centrifuged for 10 minutes at 14,000 *g* and 4℃, after which 400 μL of the supernatant was transferred to a vial and dried. The dried samples were then redissolved in 150 μL methanol-water (in a 1:1 volume ratio), awaiting metabolic panel analysis. Another 100 μL plasma sample was mixed with 300 μL pre-cooled methanol, which included the mixed internal standard (Lyso PC17.0, cholic acid-D4) at a concentration of 4 μg/mL. The mixture was vortexed for 1 minute. Subsequently, 1 mL of MTBE (methyl tert-butyl ether) and 200 μL of ultra-pure water were added stepwise and vortexed, followed by incubation at room temperature for 30 minutes. After centrifugation at 14,000 *g* at 4℃ for 15 minutes, the upper organic phase was collected in a new centrifuge tube. The lower layer underwent two extractions using 400 μL methyl tert-butyl ether (MTBE)/methanol/water (12:4:3, v/v/v). The mixed organic phases were then dried under nitrogen gas. The dried extract was redissolved in 150 μL of isopropyl alcohol/acetonitrile/water solution (7:2.5:0.5), awaiting lipidomic analysis. Finally, the samples were centrifugated at 4℃ for 10 minutes at 14,000 *g*, and the supernatant was transferred into the insert of an LC-MS injection vial for LC-MS/MS analysis. The quality control (QC) sample was prepared by mixing extracts from all samples in equal volumes.

***Instrumental analysis***

Metabolomics analysis was conducted on a Waters ACQUITY UPLC I-Class (Waters Corporation, Milford, MA, USA) in tandem with a Thermo Q Exactive Plus (Thermo Fisher Scientific, Waltham, MA, USA). Chromatography was conducted using an ACQUITY UPLC HSS T3 column (100 mm×2.1 mm, 1.8 μm) maintained at 45℃. The mobile phase consisted of water (containing 0.1% formic acid) as phase A and acetonitrile (containing 0.1% formic acid) as phase B, flowing at a rate of 0.35 mL/min. A 5 μL sample was injected for analysis. For lipidomics analysis, Dionex Ultimate 3000 UHPLC tandem Q Exactive Plus (Thermo Fisher Scientific, Waltham, MA, USA) was employed. Chromatography utilized an ACQUITY UPLC BEH C8 column (100 mm × 2.1 mm, 1.7 μm) maintained at 55℃. The mobile phase A (acetonitrile: water = 6:4) contained 10 mmol/L ammonium acetate, while mobile phase B (isopropyl alcohol: acetonitrile = 9:1) also contained 10 mmol/L ammonium acetate, flowing at a rate of 0.26 mL/min. Similarly, a 5 μL sample was injected for analysis. The data were acquired in both positive and negative ionization modes respectively. QC samples were analyzed at the beginning and the end of each batch to monitor instrument stability and injected once every 8 samples to minimize the remnants' effects during the whole sequence injection process to monitor the stability throughout the analysis. All the samples for analysis were injected randomly throughout the experiment.

***MS raw data analysis***

The raw format files of metabolomics exported from Q Exactive were processed using the metabolomics software Progenesis QI (v. 3.0, Waters Corporation, Milford, MA, USA) for baseline filtering, peak identification, integration, retention time correction, peak alignment, and normalization. Metabolite identification was based on multiple dimensions, including retention time (RT), accurate mass, secondary fragments, and isotopic distribution, and was analyzed using the Human Metabolome Database (HMDB), Lipidmaps (v2.3), METLIN database, and a local database (LuMet-Animal3.0). The error threshold for precursor ion matching was set at 5 ppm (HMDB + Lipidmaps)/20 ppm (LuMet-Animal + METLIN), and the threshold for fragment ion matching was set at 10 ppm (HMDB + Lipidmaps)/20 ppm (LuMet-Animal + METLIN). The extracted data underwent missing value processing, score filtering, and data merging. Missing value and zero-value replacement: Ion peaks with missing values >50% within a group were removed, and the remaining missing values were replaced with half of the minimum ion intensity across all samples.

The LipidSearch software (Thermo Fisher Scientific, Waltham, MA, USA) was employed to process the raw data exported from the Q Exactive Plus. Based on the precursor ions and multi-stage mass spectrometry data from each individual sample, the lipid molecular structures and their adduct patterns in positive and negative ions were identified. The search results for each independent sample were aligned within a specified retention time window. For each sample, the peak signal intensities were normalized to the total peak area, and ion peaks with missing values exceeding 50% in any group of samples were removed. The remaining missing values were replaced with half of the minimum value. The positive and negative ion data were merged into a single data matrix table, encompassing all the information extracted from the raw data that could be utilized for analysis.

The information comparison of ion fragments utilized the LipidSearch software, Human Metabolome Database (HMDB), Lipidmaps (v. 2.3), METLIN and LuMet-Animal (v. 3.0) databases. The relative standard deviation (RSD) of the metabolites in QC samples was calculated. Metabolites with RSD >30% were excluded from all the samples. The positive and negative ion data were merged into a single data matrix table for further analysis.

**Supplementary** **Table 1: Summary of data completeness for clinical indicators**

|  | Discovery cohort | | | | |  | | | | Validation cohort | | | |
| --- | --- | --- | --- | --- | --- | --- | --- | --- | --- | --- | --- | --- | --- |
| Indicator | N.  Pre-BPA | | | N. Post-BPA | | N. missing | | | | N.  Pre-BPA | N. Post-BPA | N. missing | |
|  |  |  |  |  |  | Pre-BPA | | Post-BPA | |  |  | Pre-BPA | Post-BPA |
| Age | | 65 | 65 | | 0 | | 0 | | 17 | | 17 | 0 | 0 |
| Female | | 48 | 48 | | 0 | | 0 | | 12 | | 12 | 0 | 0 |
| Male | | 17 | 17 | | 0 | | 0 | | 5 | | 5 | 0 | 0 |
| Times | | 65 | 65 | | 0 | | 0 | | 17 | | 17 | 0 | 0 |
| BMI | | 65 | 65 | | 0 | | 0 | | 17 | | 17 | 0 | 0 |
| 6MWD | | 59 | 59 | | 6 | | 6 | | 16 | | 16 | 1 | 1 |
| DDimer | | 64 | 64 | | 1 | | 1 | | 12 | | 12 | 5 | 5 |
| NTproBNP | | 64 | 64 | | 1 | | 1 | | 17 | | 17 | 0 | 0 |
| PASP | | 53 | 53 | | 12 | | 12 | | 17 | | 17 | 0 | 0 |
| TRV | | 50 | 50 | | 15 | | 15 | | 17 | | 17 | 0 | 0 |
| RA.area | | 63 | 63 | | 2 | | 2 | | 17 | | 17 | 0 | 0 |
| RVD | | 64 | 64 | | 1 | | 1 | | 17 | | 17 | 0 | 0 |
| S’ | | 64 | 64 | | 1 | | 1 | | 17 | | 17 | 0 | 0 |
| LVEDD | | 64 | 64 | | 1 | | 1 | | 17 | | 17 | 0 | 0 |
| LVEF | | 64 | 64 | | 1 | | 1 | | 17 | | 17 | 0 | 0 |
| EI | | 39 | 39 | | 26 | | 26 | | 16 | | 16 | 1 | 1 |
| TAPSE | | 64 | 64 | | 1 | | 1 | | 17 | | 17 | 0 | 0 |
| SBP | | 64 | 64 | | 1 | | 1 | | 17 | | 17 | 0 | 0 |
| DBP | | 64 | 64 | | 1 | | 1 | | 17 | | 17 | 0 | 0 |
| sPAP | | 64 | 64 | | 1 | | 1 | | 17 | | 17 | 0 | 0 |
| mPAP | | 65 | 65 | | 0 | | 0 | | 17 | | 17 | 0 | 0 |
| dPAP | | 64 | 64 | | 1 | | 1 | | 17 | | 17 | 0 | 0 |
| mPAWP | | 31 | 31 | | 34 | | 34 | | 16 | | 16 | 1 | 1 |
| CO | | 45 | 45 | | 20 | | 20 | | 16 | | 16 | 1 | 1 |
| CI | | 45 | 45 | | 20 | | 20 | | 16 | | 16 | 1 | 1 |
| SaO_2_ | | 63 | 63 | | 2 | | 2 | | 17 | | 17 | 0 | 0 |
| PaCO_2_ | | 56 | 56 | | 9 | | 9 | | 17 | | 17 | 0 | 0 |
| PVR | | 45 | 45 | | 20 | | 20 | | 16 | | 16 | 1 | 1 |
| SvO_2_ | | 64 | 64 | | 1 | | 1 | | 17 | | 17 | 0 | 0 |
| TAPSE/sPAP | | 63 | 63 | | 2 | | 2 | | 17 | | 17 | 0 | 0 |

6MWD: 6-min walking distance; BMI: Body mass index; BPA: Balloon pulmonary angioplasty; CI: Cardiac index; CO: Cardiac output; DBP: Diastolic blood pressure; dPAP: Diastolic pulmonary artery pressure; EI: Eccentricity index; LVEDD: Left ventricular end-diastolic diameter; LVEF: Left ventricular ejection fraction; mPAP: Mean pulmonary artery pressure; mPAWP: Mean pulmonary artery wedge pressure; NT-proBNP: N-terminal pro B-type natriuretic peptide; PASP: Pulmonary artery systolic pressure; PVR: Pulmonary vascular resistance; RA area: Right atrial area; RVD: Right ventricular basal diameter; S’: Tricuspid annular peak systolic velocity; SaO_2_: Arterial oxygen saturation; SBP: Systolic blood pressure; sPAP: Systolic pulmonary artery pressure; SvO_2_: Mixed venous oxygen saturation; TAPSE: Tricuspid annular plane systolic excursion; TRV: Tricuspid regurgitation velocity.

**Supplementary Table 2: Baseline characteristics of CTEPH participants.**

| **Items** |  | **Discovery cohort** | | |  | | **Validation cohort** | | | |  |  |
| --- | --- | --- | --- | --- | --- | --- | --- | --- | --- | --- | --- | --- |
| **Number of subjects** | | 65 | | | |  | | 17 | | | | |
| **Demographics** | |  | |  | |  | |  | |  | | |
| Age, years | | 66.0 (59.0-69.0) | | | |  | | 69.0 (50.0-84.0) | | | | |
| Gender | Female | 48 (74) | | | |  | | 12 (70) | | | | |
|  | Male | 17 (26) | | | |  | | 5 (30) | | | | |
| Number of BPA sessions | | 4.0 (4.0-5.0) | | | |  | | 4.0 (4.0-5.0) | | | | |
| **Medication** | |  |  | |  | |  | |  | | |  |
| Riociguat |  | 49 (76) | | |  | | 14 (82) | | | | |  |
| Ambrisentan |  | 12 (18) | | |  | | 1 (6) | | | | |  |
|  | Rivaroxaban | 35 (54) | | |  | | 4 (24) | | | | |  |
| Anticoagulation | Edoxaban | 14 (22) | | |  | | 9 (52) | | | | |  |
|  | Warfarin | 16 (24) | | |  | | 4 (24) | | | | |  |

Data are presented as *n*, *n* (%) or median (interquartile range). CTEPH: Chronic thromboembolic pulmonary hypertension; BPA: Balloon pulmonary angioplasty.

**Supplementary Table 3: Clinical indicators of CTEPH participants who received BPA treatment.**

|  |  | **Discovery cohort** | |  | | **Validation cohort** | | |  | |  |
| --- | --- | --- | --- | --- | --- | --- | --- | --- | --- | --- | --- |
|  |  | **Pre-BPA** | **Post-BPA** | | **Sig.** | | **Pre-BPA** | **Post-BPA** | | **Sig.** | |
| Body mass index, kg/m^2^ | | 23.7 (22.3-26.3) | 24.3 (22.5-26.4) | | NS | | 23.5 (21.7-25.8) | 23.4 (21.8-25.3) | | NS | |
| 6-min walking distance, m | | 340.0 (242.5-400.0) | 460.0 (412.5-501.0) | | *** | | 394.5 (266.3-401.3) | 437.5 (373.8-470.0) | | ** | |
| D-Dimer, ng/mL | | 0.20 (0.07-0.37) | 0.11 (0.06-0.23) | | ** | | 0.45 (0.17-0.94) | 0.24 (0.13-0.39) | | NS | |
| NT-proBNP, ng/L | | 630.7 (126.9-1698.5) | 99.5 (52.9-166.4) | | *** | | 317.6 (79.5-1614.0) | 78.0 (56.0-120.5) | | ** | |
| **Echocardiography** | |  |  | |  | |  |  | |  | |
| PASP, mmHg | | 71 (59-87) | 44 (37-50) | | *** | | 69 (59-84) | 45 (39-56) | | ** | |
| TRV, m/s | | 4.1 (3.5-4.6) | 3.0 (2.8-3.3) | | *** | | 4.0 (3.7-4.5) | 3.1 (2.7-3.6) | | *** | |
| RA area, cm² | | 18.1 (14.8-22.8) | 13.5 (11.5-16.3) | | *** | | 19.8 (16.0-22.5) | 14.5 (13.7-17.2) | | ** | |
| RVD, cm | | 4.0 (3.7-4.4) | 3.3 (2.9-3.6) | | *** | | 4.0 (3.7-4.4) | 3.4 (3.1-3.8) | | *** | |
| S’, cm/s | | 11.0 (9.4-13.0) | 12.0 (10.1-13.0) | | ** | | 10.0 (9.0-12.0) | 10.0 (9.0-12.0) | | NS | |
| LVEDD, cm | | 4.2 (3.9-4.7) | 4.7 (4.3-5.0) | | *** | | 4.4 (3.6-4.6) | 4.9 (4.3-5.0) | | NS | |
| LVEF, % | | 76 (71-80) | 76 (72 -80) | | NS | | 78 (72-84) | 77 (68-83) | | NS | |
| EI | | 1.23 (1.10-1.45) | 1.00 (1.00-1.02) | | *** | | 1.18 (1.00-1.26) | 1.00 (1.00-1.01) | | ** | |
| TAPSE, mm | | 19.0 (16.8-21.0) | 20.0 (18.7-23.0) | | *** | | 19.0 (16.0-22.0) | 21.0 (19.0-22.0) | | NS | |
| TAPSE/sPAP | | 0.22 (0.17-0.27) | 0.46 (0.39-0.53) | | *** | | 0.24 (0.19-0.28) | 0.45 (0.32-0.60) | | *** | |
| **Haemodynamics** | |  |  | |  | |  |  | |  | |
| SBP, mmHg | | 120 (110-127) | 115 (106-120) | | * | | 105 (95-121) | 109 (99-119) | | NS | |
| DBP, mmHg | | 72 (64-82) | 69 (63-73) | | ** | | 69 (59-73) | 63 (56-70) | | NS | |
| sPAP, mmHg | | 85 (72-101) | 45 (39-51) | | *** | | 90 (66-96) | 47 (40-62) | | *** | |
| mPAP, mmHg | | 43 (38-52) | 26 (22-28) | | *** | | 45 (31-49) | 22 (20-32) | | *** | |
| dPAP, mmHg | | 23 (18-27) | 12 (10-15) | | *** | | 24 (18-26) | 11 (8-15) | | *** | |
| mPAWP, mmHg | | 7 (6-10) | 9 (6-11) | | NS | | 8 (6-9) | 7 (4-10) | | NS | |
| CO, L/min | | 4.3 (3.7-5.5) | 5.4 (4.9-6.1) | | *** | | 3.7 (3.2-5.5) | 5.4 (4.7-6.3) | | * | |
| CI, L/min/m² | | 2.7 (2.2-3.1) | 3.3 (2.9-3.8) | | *** | | 2.3 (2.1-2.9) | 3.3 (3.0-3.5) | | * | |
| SaO_2_, % | | 93.5 (90.6-95.0) | 95.5 (93.8-97.3) | | *** | | 92.2 (90.6-93.7) | 94.2 (93.0-96.5) | | ** | |
| PVR, Wood unit | | 8.25 (6.47-11.29) | 2.83 (2.45-3.44) | | *** | | 8.86 (5.62-12.74) | 3.25 (2.53-4.21) | | *** | |
| SvO_2_, % | | 61.7 (56.2-65.4) | 68.5 (66.1-73.6) | | *** | | 60.9 (56.2-64.9) | 68.3 (66.40-72.9) | | *** | |

Data are presented as *n*, *n* (%) or median (interquartile range). Statistical comparisons were conducted using either a paired samples *t*-test when both pre- and post-PBA groups exhibited normal distribution (Shapiro-Wilk test *P* ≥0.05) or a Wilcoxon signed-rank test when either group deviated from normality (Shapiro-Wilk test *P* <0.05). CTEPH: Chronic thromboembolic pulmonary hypertension; BPA: Balloon pulmonary angioplasty; NT-proBNP: N-terminal pro B-type natriuretic peptide; PASP: Pulmonary artery systolic pressure; TRV: Tricuspid regurgitation velocity; RA area: Right atrial area; RVD: Right ventricular basal diameter; S’: Tricuspid annular peak systolic velocity; LVEDD: Left ventricular end-diastolic diameter; LVEF: Left ventricular ejection fraction; EI: Eccentricity index; TAPSE: Tricuspid annular plane systolic excursion; SBP: Systolic blood pressure; DBP: Diastolic blood pressure; sPAP: Systolic pulmonary artery pressure; mPAP: Mean pulmonary artery pressure; dPAP: Diastolic pulmonary artery pressure; mPAWP: Mean pulmonary artery wedge pressure; CO: Cardiac output; CI: Cardiac index; SaO_2_: Arterial oxygen saturation; SvO_2_: Mixed venous oxygen saturation; PVR: Pulmonary vascular resistance; Sig.: Significance; NS: Not significant; ^*^*P* <0.05; ^**^*P* <0.01; ^***^*P* <0.001.


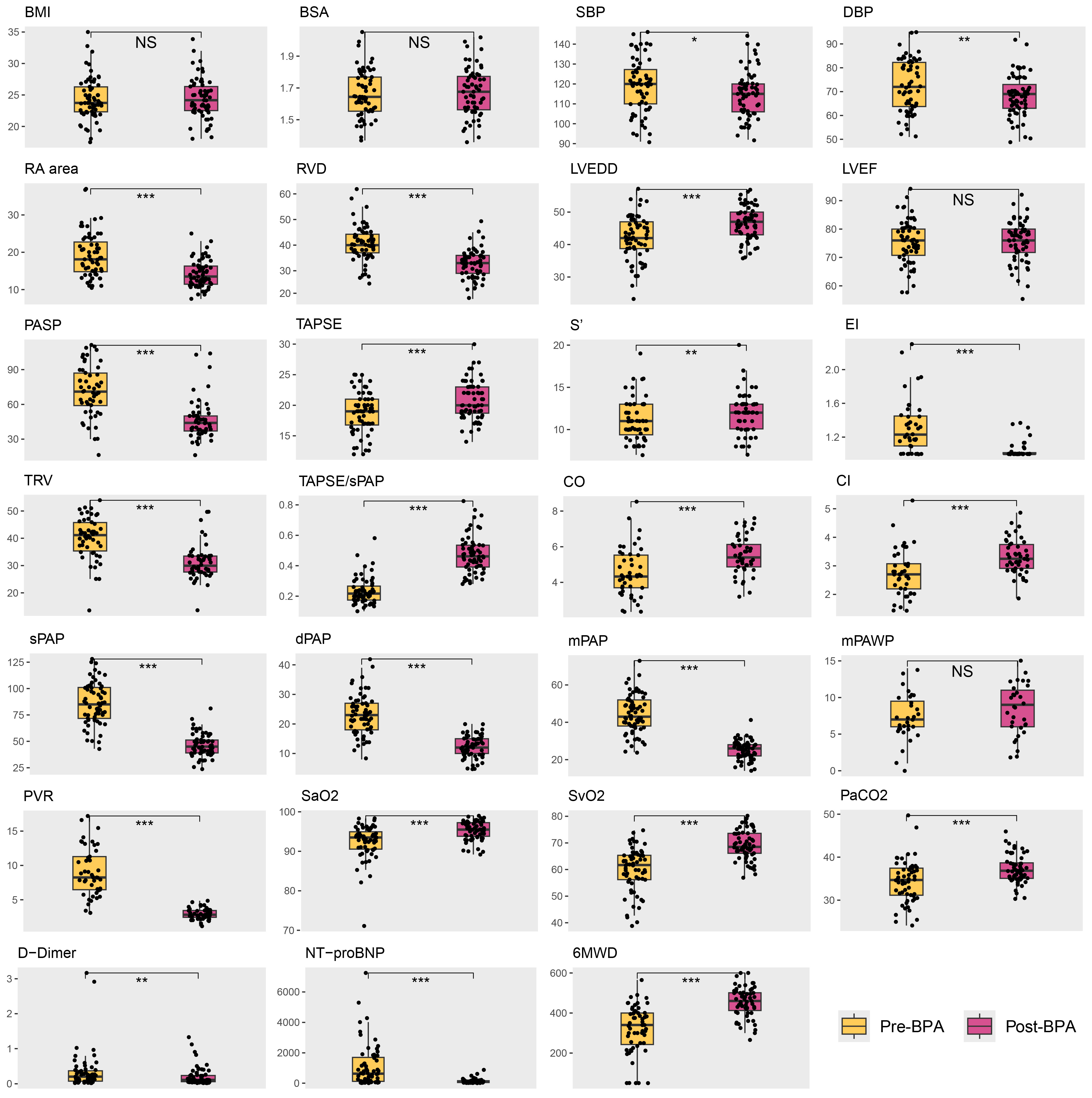


**Supplementary Fig. 1. Box-dot plots comparing clinical indicators in CTEPH patients before and after BPA treatment (discovery cohort, *n*=65).** Statistical comparisons were performed using paired samples *t*-tests for normally distributed data (Shapiro-Wilk test *P* ≥0.05) or Wilcoxon signed-rank tests for non-normally distributed data (Shapiro-Wilk test *P* < 0.05). **P* <0.05; ***P* <0.01; ****P* <0.001. NS: not significant.
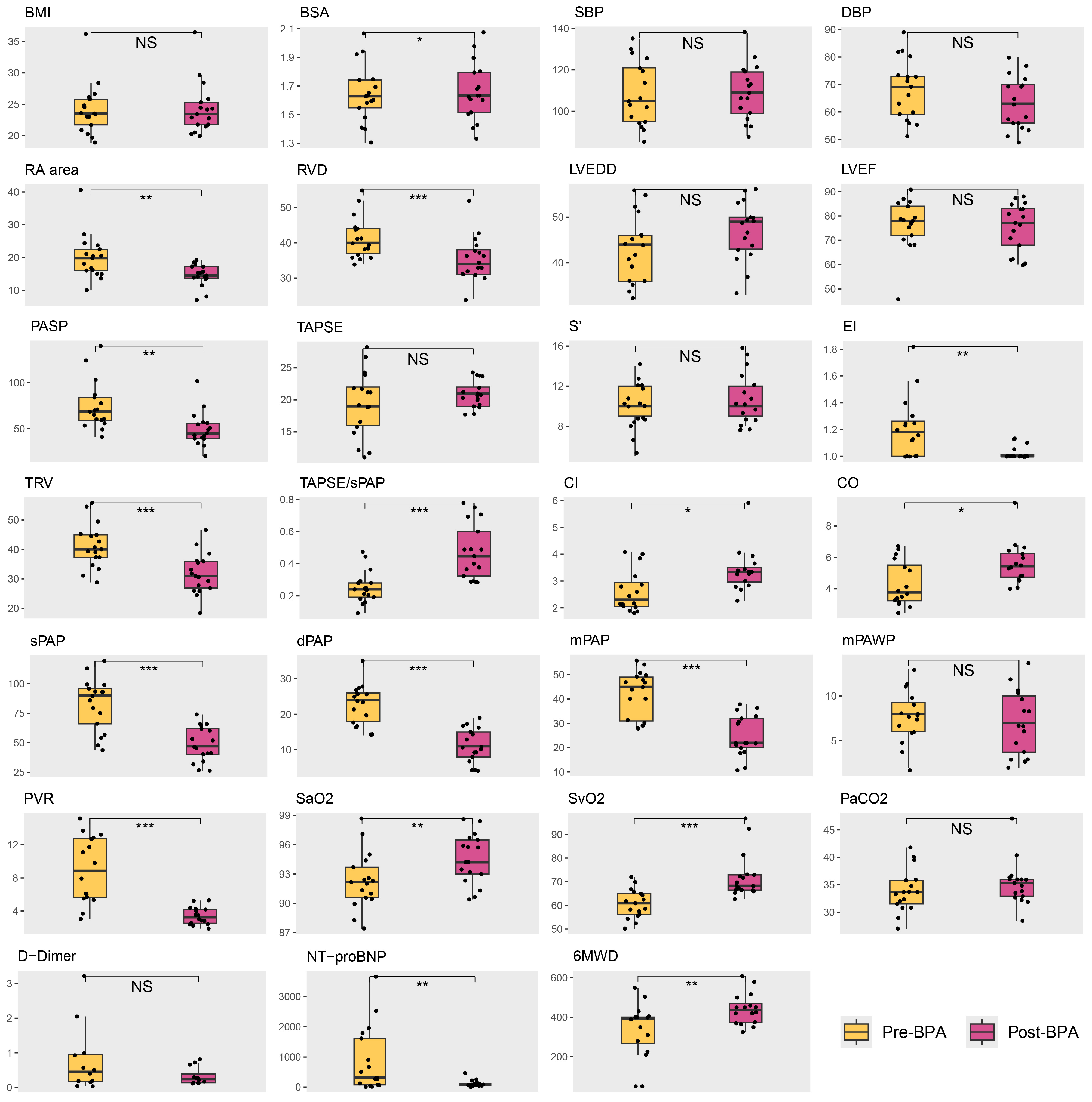


**Supplementary Fig. 2. Box-dot plots comparing clinical indicators in CTEPH patients before and after BPA treatment (validation cohort, *n*=17).** Statistical comparisons were performed using paired samples *t*-tests for normally distributed data (Shapiro-Wilk test *P* ≥0.05) or Wilcoxon signed-rank tests for non-normally distributed data (Shapiro-Wilk test *P* <0.05). **P* <0.05; ***P* <0.01; ****P* <0.001. NS: not significant.


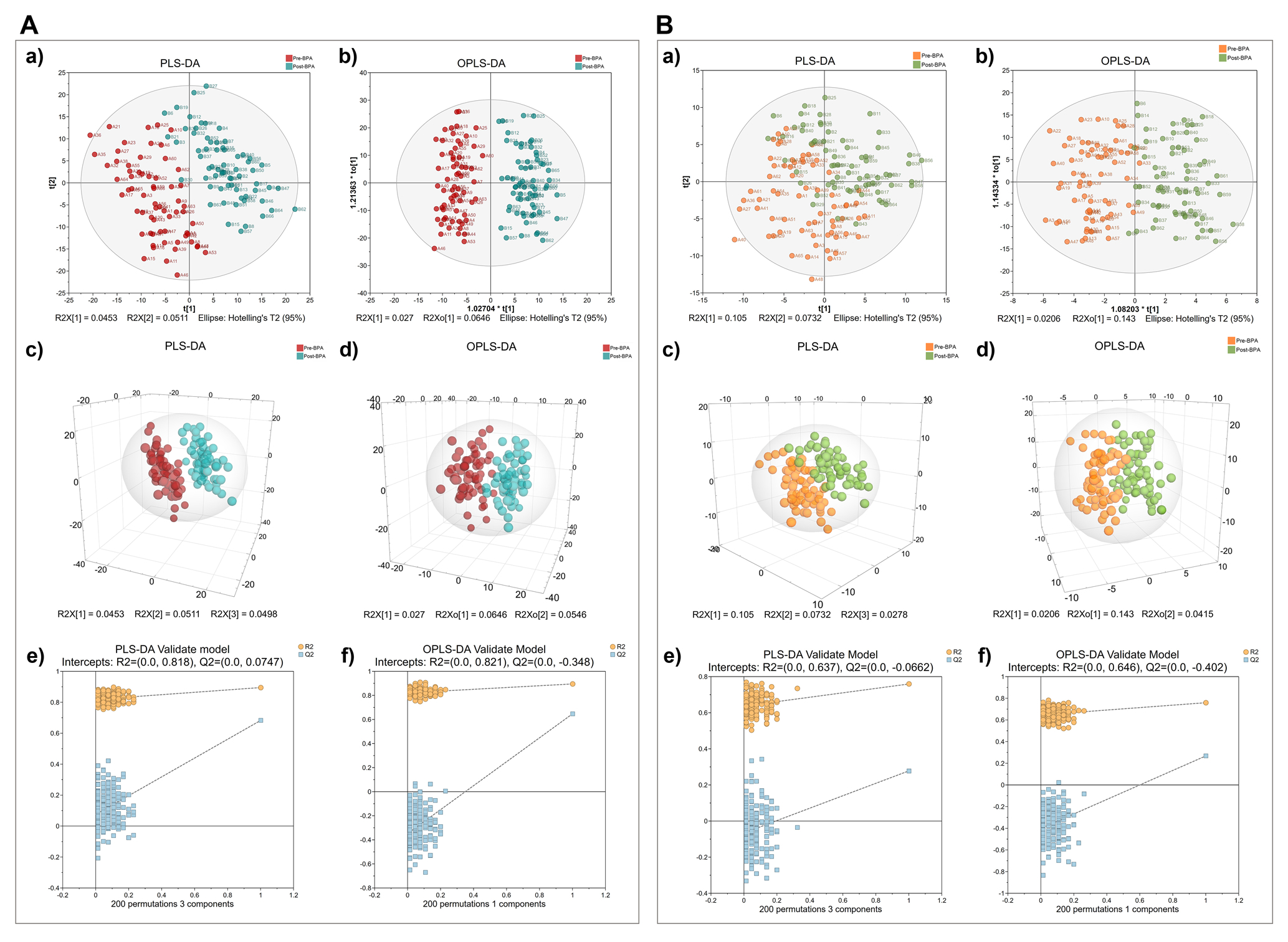


**Supplementary Fig. 3. Multivariate statistical analysis of global metabolomic or lipidomic profiles in participants with CTEPH between pre- and post-BPA.**

**Panel A**: Analysis of metabolomic profiles. **Panel B**: Analysis of lipidomic profiles. **a)**, **b)** 2D plots of partial least squares discriminant analysis (PLS-DA) and orthogonal partial least squares discriminant analysis (OPLS-DA) for pre- and post-BPA. **c)**, **d)** 3D plot of PLS-DA and OPLS-DA for pre- and post-BPA. **e)**, **f)** The 200-permutation test of each discriminant analysis.


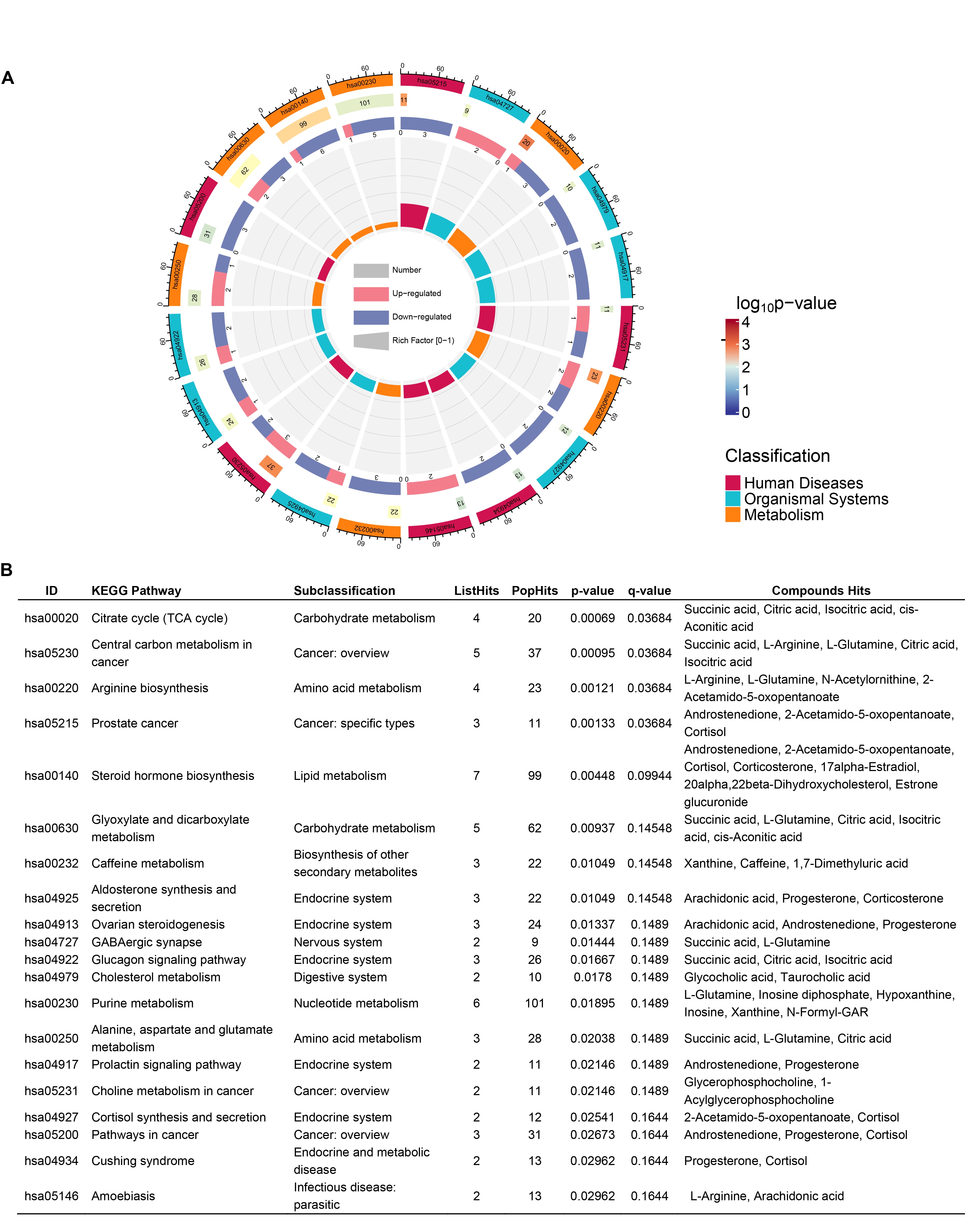


**Supplementary Fig. 4. KEGG pathway enrichment analysis using the differential metabolites. (A)** Circle diagram of KEGG pathway enrichment analysis of differential metabolites. (**B)** The detailed information of each enriched pathway.

**
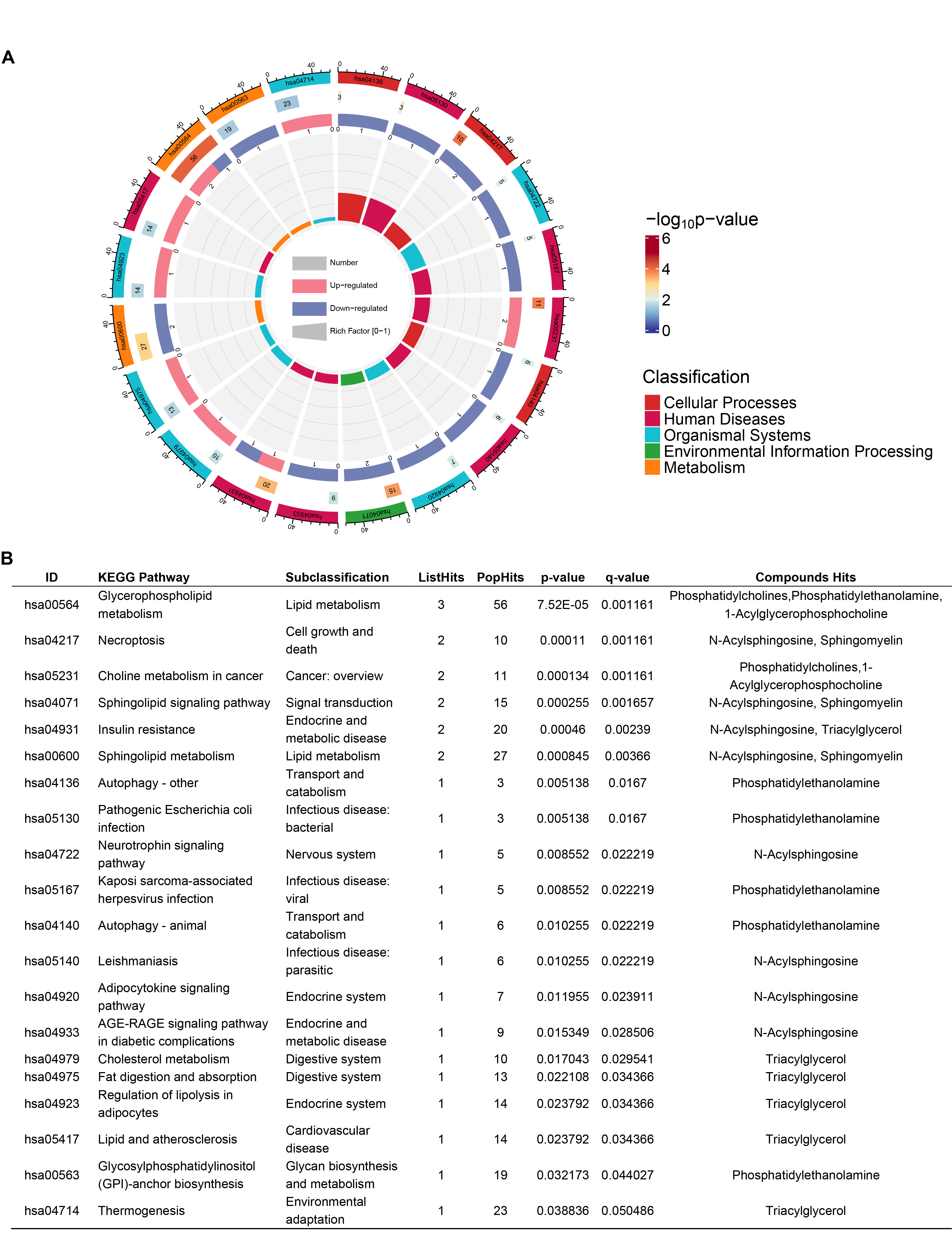
**

**Supplementary Fig. 5. KEGG pathway enrichment analysis using the differential lipids. (A)** Circle diagram of KEGG pathway enrichment analysis of differential lipids. (**B)** The detailed information of each enriched pathway.


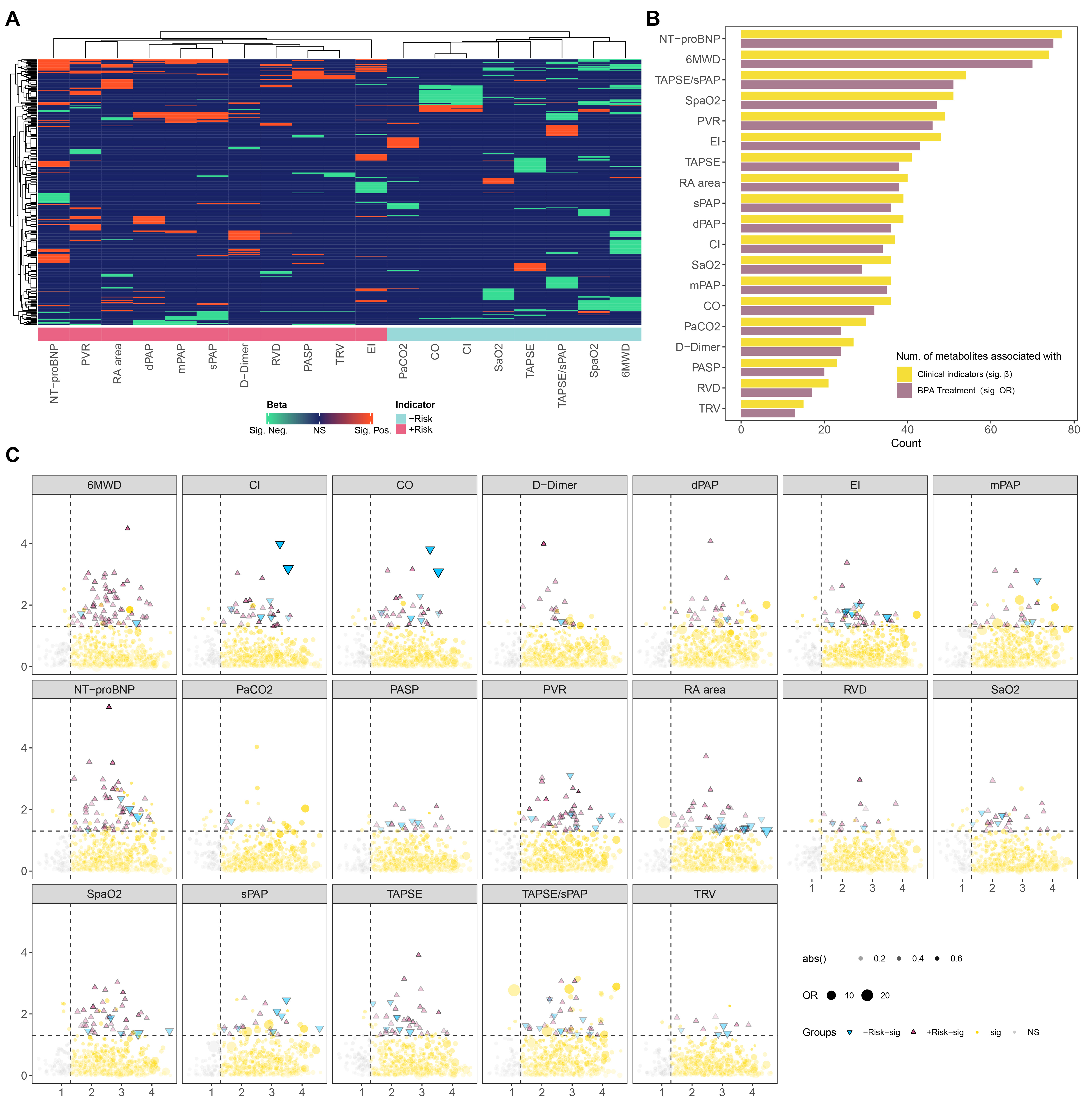


**Supplementary Fig. 6. Associations of differential compounds (metabolites and lipids) with clinical indicators and treatment status.**

**(A)** Heatmap illustrating the associations between standardized changes in clinical indicators and log_2_FC in metabolites following BPA treatment. Each cell in the heatmap represents the adjusted regression coefficient derived from a multiple linear regression. Cells are colored red (positive) or green (negative) to indicate statistical significance and directionality. (**B)** Bar chart summarizing the number of metabolites significantly associated *(P* <0.05) with each clinical indicator (e.g., mPAP, 6MWD) and their concurrent associations with BPA treatment status (pre- *vs*. post-BPA) in 65 CTEPH patients. (**C)** Scatter plot depicting associations of metabolites with treatment status (pre- *vs*. post-BPA) and clinical indicators. The x-axis shows *P* values from adjusted odds ratios (conditional logistic regression, assessing treatment response). The y-axis shows *P* values from adjusted β coefficients (multiple linear regression, assessing association with indicators). Metabolites are classified as favorable (blue downward triangles, positive with treatment response and favorable outcomes or negative with adverse outcomes), unfavorable (red upward triangles, negative with treatment response and favorable outcomes or positive with adverse outcomes), and other significant compounds (gold circles, significant but not conforming to favorable/unfavorable patterns; *P* <0.05). Models adjusted for BPA session count, age, sex, body mass index (BMI), and body surface area (BSA).


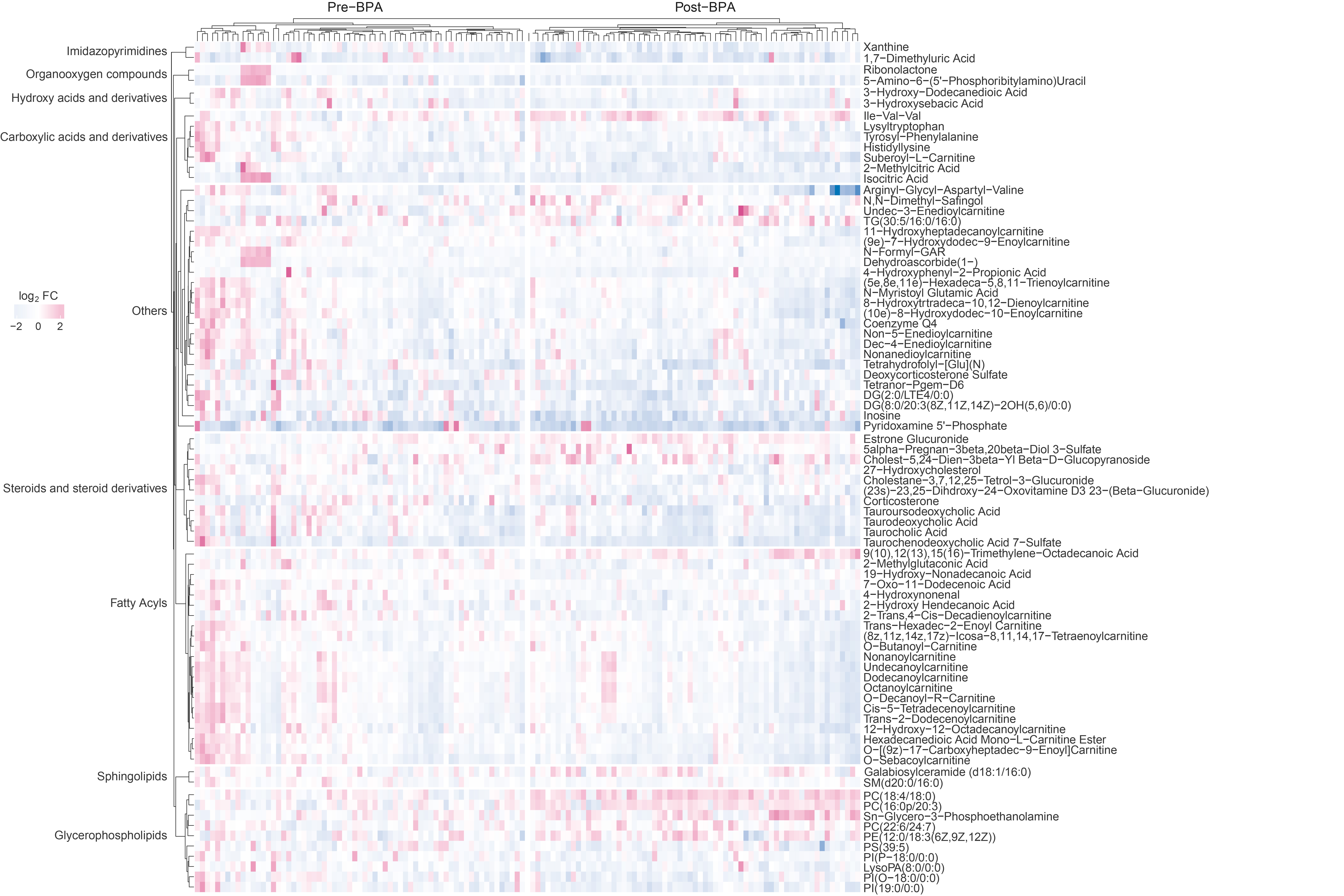


**Supplementary Fig. 7. Heatmap of log_2_FC of the 81 compounds significantly associated with the clinical indicators.**

Pink blocks indicate metabolites with increased levels, while light blue blocks represent those with decreased levels. DG: Diglyceride; LysoPA: Lysophosphatidic acid; PC: Phosphatidylcholine; PE: Phosphatidylethanolamine; PI: Phosphatidylinositol; PS: Phosphatidylinositol; SM: Sphingomyelin; TG: Triglyceride.
